# Emergency surgery in the elderly: the balance between function, frailty, fatality and futility

**DOI:** 10.1186/s13049-015-0099-x

**Published:** 2015-02-03

**Authors:** Kjetil Søreide, Kari F Desserud

**Affiliations:** Department of Gastrointestinal Surgery, Stavanger University Hospital, Stavanger, Norway; Department of Clinical Medicine, University of Bergen, Bergen, Norway

**Keywords:** Emergency surgery, Mortality, Morbidity, Geriatric medicine, Health care systems, Organization, Outcomes

## Abstract

Becoming old is considered a privilege and results from the socioeconomic progress and improvements in health care systems worldwide. However, morbidity and mortality increases with age, and even more so in acute onset disease. With the current prospects of longevity, a considerable number of elderly patients will continue to live with good function and excellent quality of life after emergency surgical care. However, mortality in emergency surgery may be reported at 15-30%, doubled if associated with complications, and notably higher in patients over 75 years. A number of risks associated with death are reported, and a number of scores proposed for prediction of risk. Frailty, a decline in the physiological reserves that may make the person vulnerable to even the most minor of stressful event, appears to be a valid indicator and predictor of risk and poor outcome, but how to best address and measure frailty in the emergency setting is not clear. Futility may sometimes be clearly defined, but most often becomes a borderline decision between ethics, clinical predictions and patient communication for which no solid evidence currently exists. The number and severity of other underlying condition(s), as well as the treatment alternatives and their consequences, is a complex picture to interpret. Add in the onset of the acute surgical disease as a further potential detrimental factor on function and quality of life – and you have a perfect storm to handle. In this brief review, some of the challenging aspects related to emergency surgery in the elderly will be discussed. More research, including registries and trials, are needed for improved knowledge to a growing health care challenge.

## Background

In a couple of years the number of people aged ≥65 years will outnumber the number of people ages ≤5 years or less – globally [1]. One may think that the growth in the elderly population is predominantly seen in developed countries, but the number of elderly people is increasing fastest in countries belonging to the low- and middle-income countries (LMICs). Thus, caring for the elderly population also from a health perspective is a global responsibility and is of worldwide interest. Indeed, becoming old is considered a privilege and results from the socioeconomic development and improvements in health care systems worldwide.

**Figure 1:**
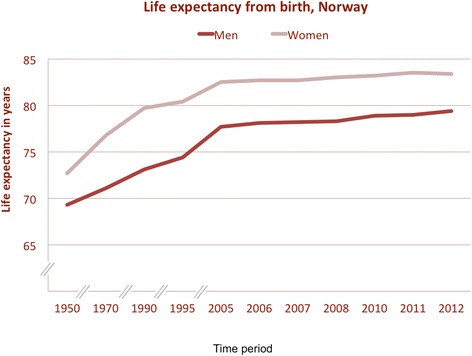
**Life-expectancy at birth in Norway.** Data based on numbers from Statistics Norway (http://www.ssb.no).

While the current life-expectancy at birth is unsurpassed in human history (Figure 1), it is maybe even more important to note the life expectancy of a 65 year old, is currently estimated at 85–90 years in western countries (such as US, UK, Scandinavia). Thus, an acute medical insult may thus deprive a healthy 65- or 75-years old person from a considerable numbers of future life-years (20–30 years), either as lived in dependency (if severe morbidity follows) or years lost (if death ensues). This has consequences for both the individual patient, but also for society as such.

In particular, as the population grows older, the number of added organs with disease conditions that may need treatment or support become more prevalent [2], yet also with an increasing associated additional risk burden by surgery. This burden may be related to comorbidity – often in the form of kidney disease, heart disease, or lung disease - that may require particular attention. Will the patient need dialysis? Will the heart tolerate the surgical stress? Will the pulmonary condition require ventilator support, and, if yes, when will the patient be able to be weaned off the ventilator, if at all? Clearly, the number and severity of other underlying condition(s), as well as the treatment alternatives and their consequences, is a complex picture to handle. Add in the impact on the acute surgical disease on further function and quality of life – and you have a perfect storm to handle. In this brief review, some of the challenging aspects related to emergency surgery will be discussed.

### Growing population of elderly

**Figure 2:**
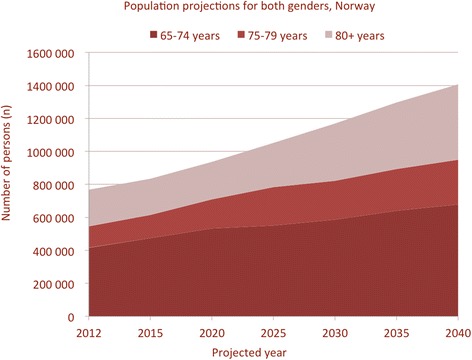
**Projected number of elderly per age-group and both genders.** Data from Folkehelseinstituttet (copyright Norwegian Institute of Public Health) and produced with permission.

The number of elderly people will increase dramatically over the next few decades (Figure 2), with population projections towards 2040 indicating a 66% increase in the age-groups 65 to 74 years [3]. More importantly, the age groups 75 years and above are projected to increase with >100%, which clearly will have implications for future health services. Also, with increasing age comes an added risk of additional disease as well as the use of drugs, some of which clearly can interfere with emergency surgical conditions [4].

### Organization of emergency surgical care

Emergency surgical services have improved from structural changes and clear management pathways over the past recent years [5,6]. The trauma community, hit by the wave of elderly injured patients already, has started to organize focused multidisciplinary services that focus on the pathway of care that incorporates the geriatric considerations [7-9].

In Scandinavia, the evolving attention of emergency care has not been as clear, and emergency surgery still falls within the realm of the ‘general surgeon’, a vanishing breed, as previously discussed in this journal [10]. Restructuring of emergency surgery services has not seen a change yet, although still heavily debated. Notably, as surgery is getting more subspecialized, the emergency surgical care becomes more fractioned, with no or little dedicated focus to the overall health care burden associated with emergency surgery conditions [11]. As it has been demonstrated that the overall exposure to emergency surgery is low in many hospitals in Scandinavia [12], and that the overall operative volume is very low, one may question the separation of services into disease-oriented or specialty-oriented functions.

Notably, for more complex disorders it is many times hard to implement change that demonstrable leads to better outcomes [13], as is the case for surgery in several instances. As demonstrated in other medical fields – such as cancer care - the impact of multidisciplinary teams is necessary for complex evaluation and decision-making. Geriatric emergency surgery behoves the recognition of multidisciplinary approach, including geriatric specialists in the overall work-up and treatment planning together with the surgeon. Geriatric consultation has been demonstrated for hip fracture care and trauma patients with improvements in outcome [9,14]. The problem remains in access to and the number of geriatric specialists, which is a limited resource in most hospitals.

### Strategies for optimal care

**Figure 3:**
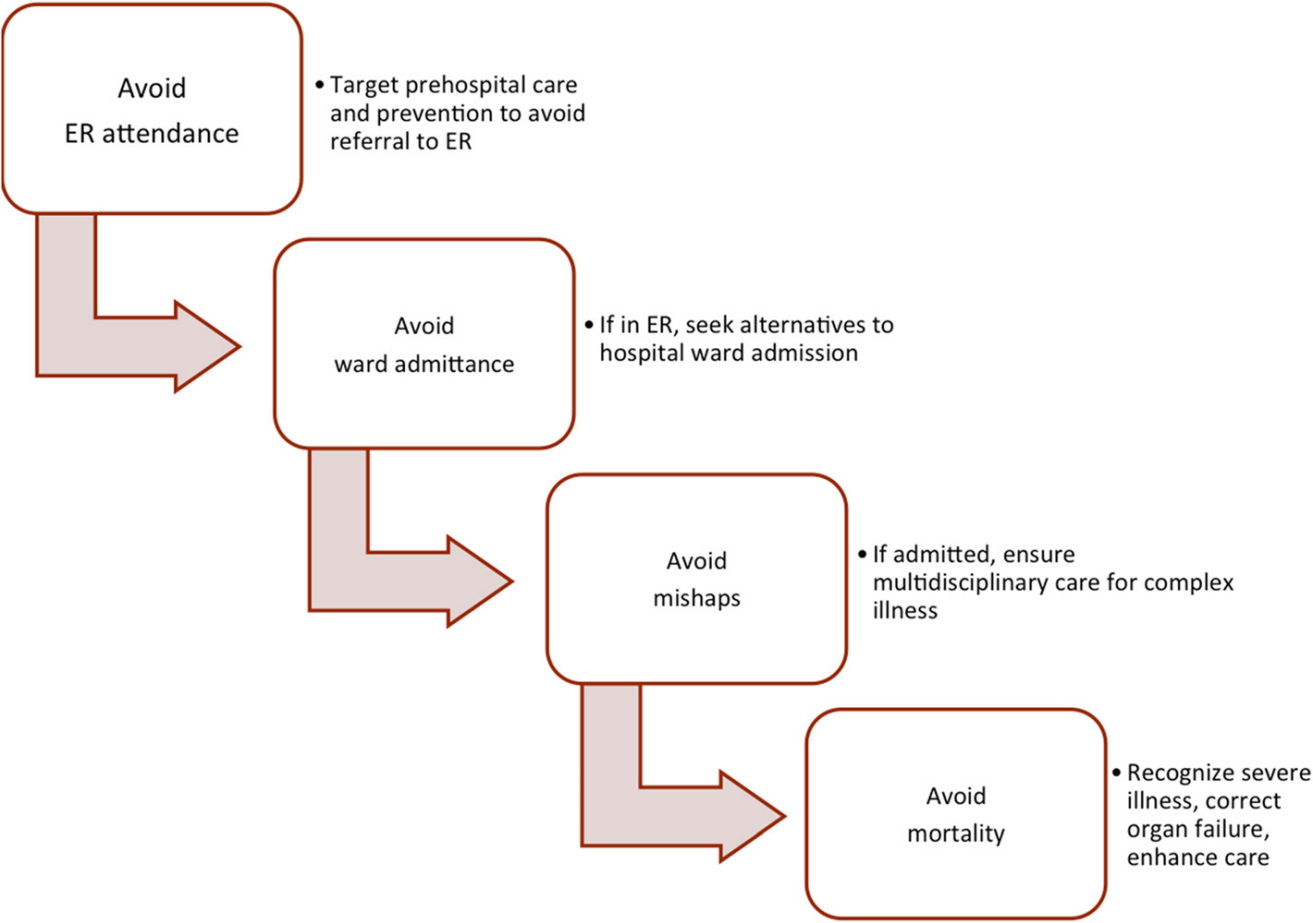
**Steps to consider in optimal care pathways as framework for clinical care improvement and research targets.**

Unnecessary admissions and over treatment is one of the major pitfalls in the care of the elderly, but can be avoided by establishing appropriate frameworks for clinical care pathways and research [15] (Figure 3). Emergency admissions to hospital may often occur during out-of-office periods, weekends and nights and with the risk of not having a well-defined plan. Thus, in order to minimize the burden of suffering on the patient as well as the load on the health care system, and optimalized care pathway should be scrutinized according to the available health care system resources (Figure 3).

Preferably, before admitting the patient, the primary care provider should consider if the patient is best served with outpatient treatment or by admission, especially if the symptoms is part of a chronic deterioration and not acute. For the general practitioner (GP) it can sometimes be difficult to determine if a condition is in need of acute surgery and, in practice, it is often necessary with additional examinations for accurate diagnostics. However, if the patient is unlikely to benefit from, or even tolerate, any major or minor surgical intervention, one should seriously consider other treatment options and if these can be initiated and managed out of hospital. However, the indication for surgery or not may not be clear to the primary care physician, and specialist consultation should be the next step to discuss pros and cons, treatment alternatives and possible timing of admission. Obviously, the specialist surgeon relies just as much on the expert knowledge of the referring physician to arrive at a reasonable decision, and this is always a two-way communication and decision-making. In emergency settings, it is difficult to make a qualified decision without adequate knowledge of the patient’s medical history and habitual level of function, as high age is not itself a good predictor. Emergency surgery may often be performed as a lifesaving procedure, and delay to surgery can reduce overall outcome. In the case of severely injured geriatric trauma patients, it is recommended that most patients receive aggressive care during the initial phase of treatment, as most outcomes are favourable [16]. It is also recommended, that providers establish treatment goals early [16], and the objective should in some cases be to maintain quality of life, and to avoid unnecessary and non-beneficial treatment. Last, but not least, it is absolutely necessary to include the patient and next-of-kin in the discussion, as the wishes, views and expectations may be different from that of the caretaker.

Elderly who receive acute surgery often survives the initial treatment, but often suffers from severe complications due to comorbidity. It is important with close post-operative follow up to avoid life threatening complicating conditions, and to involve geriatric consultants and other specialities if needed. Additional surgery and aggressive life-prolonging care, can in some cases, do more harm than good. Patients with end-stage cancer who is in need of acute surgery, may often be treated by the least invasive means, because of limited expected lifetime and, because of shared decisions made earlier. The aim of caregiving is not always to prevent death (Figure 3), but rather to relive suffering, provide good palliation and provide for optimal quality of life. Of notice, the involvement of geriatric consultation teams is associated with decreased mortality at 6 and 8 months after discharge, but no effect on length of stay, readmissions, and functional outcomes [17].

### The issue of frailty

While many elderly in the future will be healthier and live active, independent lives [18], the implications of frailty that comes with age becomes a pressing issue, as reviewed in detail by Clegg et al. [19]. Notably, the combined effects of heredity and environmental exposure with chronological age on biological changes cause a decline in the physiological reserves, which may make the person vulnerable to even the most minor of stressful event (such as an acute urinary tract infection) – leading to independence, morbidity and even death. In surgical colloquial terms, it is often stated that ‘the patient may tolerate an operation, but not a complication’. The expression indicates the limitations in elderly patients to cope with the insult on physiology that may be more easily overcome by a more healthy and younger person. This limitation of physiological reserve is in particular noticed in the emergency setting when the physiological responses are particularly challenged and when the treating clinician may have limited info and time to make the appropriate decisions [20].

Elderly people express an epidemiologically different disease pattern, such as demonstrated for long-bone fractures [21], hernias [22] or perforated gastroduodenal ulcers [23]. Also, short- and long-term outcomes from the same disease are often associated with higher mortality, increased morbidity, and consequently higher utility of intensive care resources and longer hospital stay, and even limitations in ability to live an independent life after the initial insult [22,24-27]. About half of patients aged >80 years at time of emergency surgery are still alive 3 years after surgery and, a continued health decline is expressed in most patients although ability to perform daily tasks was similar for patients at 1, 2 and 3 years of follow up [28].

### Fatality rates

**Figure 4:**
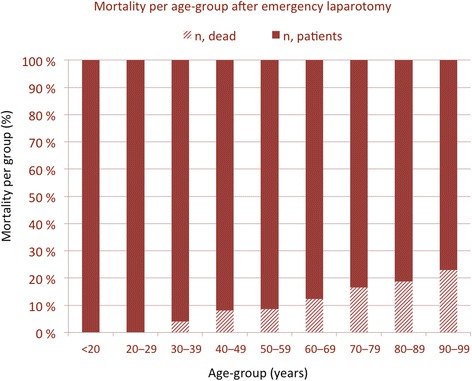
**Depicted increase in mortality rates per age group after emergency laparotomy.** Data are derived from the UK Emergency Laparotomy Network.

Overall, emergency surgery is associated with high mortality rates and remains a considerable global disease burden [29]. In the western world, the big killers are ruptured abdominal aortic aneurysms, conditions requiring emergency abdominal surgery and hip fractures [29,30]. The exact mortality rates differ between studies, based on what population is included and conditions considered. However, data from the UK Emergency Laparotomy Network [31] indicate an increased risk per age-group (Figure 4). Another UK study of nearly 370,000 emergency surgery admissions noted an overall mortality of 15,6%, with an institutional range between 9,2% and 18,2% [32]. Hospitals with lower mortality had better access to ICU beds, and higher use of CT and ultrasound investigations.

Emergency laparotomy carried a 48% mortality rate in patients aged >75 years in a Danish study [33]. In a further study, patients aged ≥90-years undergoing emergency surgery had twice the mortality rate compared to younger patients and, of notice, one-year mortality was high after both elective (29%) and emergency surgery (49%) [34]. Resectional surgery (i.e. small bowel resection) is associated with higher mortality (43%) in one study [35].

These studies point to the mortality risk with age per se, but also to the increased risk with type and invasiveness of procedure and, maybe most important, that there is considerable variation between providers of emergency surgery services.

### Futility

What defines futile care is difficult to accurately assess. One recent US study developed a prediction model based on a nationwide dataset [36]. A combination of risk factors that were associated with a <10% probability of survival, included patients age >90 years, ASA score of 5, septic shock, dependent functional status, and abnormal white blood cell count. Some have confirmed a high ASA score as predictor of mortality [37], yet others found use of vasopressors and hypoalbuminaemia as stronger predictors for mortality [26]. Notably, has also been associated with poor outcomes in elective surgery. The trouble with a low albumin level is the interpretation and relation to causality, as hypoalbumineania may indicate either a poor nutritional status; or, a catabolic disease condition; or, the presence of sepsis; or, all or some of the aforementioned in combination. Sarcopenia has similarly been associated with poor outcomes [38].

Patient preferences should preferably be discussed before an episode of acute illness occurs to allow for an individualized treatment plan with discussed and agreed limitations (if applicable) to step-up of care. This will probably reduce procedures that may prolong life, but not necessarily improve quality of life. Well-informed decisions are often more difficult to make in situations in severe distress or when surrogates need to make them ad hoc.

### Frailty

Frailty is increasingly used to describe the elderly prone to be weak or vulnerable, but the term is difficult to exactly frame. The ‘frailty phenotype’ [19] is defined by some as the presence of five criteria, including:

- unintentional weight loss,
- self-reported exhaustion,
- weakness (grip strength),
- slow walking speed, and
- low physical activity.

The themes addressed above have been incorporated into complex schemes, such as the 70-item Canadian Study of Health and Aging Frailty Index, which are unpractical or unlikely to be obtained in the emergency setting.

Although a ‘simple prognostic index’ (SPI) has been proposed for emergency laparotomy outcome prediction [39], this score has not been validated in other series, nor specifically for the elderly population. Again, a high value on comorbidity is emphasized in that score, and the difficulty in defining a universal score likely reflects the complex disease picture that is associated with acute onset disease, and as investigated for perforated ulcers [40]. A modification of the Canadian Study of Health and Aging Frailty Index in over 35,000 US emergency surgeries [41] provides further evidence for a ‘frailty index’ as an investigation of use. Notably, their modification consisted of listing 11 items, most of which are directly related to comorbid disease, and which, as such, is also expressed by the ASA score. A true advantage of this modified frailty index outside the use on large, administrative datasets in the US is thus not clear.

Notably, emergency surgery comes at a crossroads with cancer care when patient with an active cancer disease present with an emergency condition, usually in the form of a bleeding, obstruction or perforation [42]. Decision-making may then be difficult in unfit and malnourished patients [43], and besides consulting with the treating oncologist regarding prognosis (of the cancer disease seen in isolation) and further planned treatment (chemo- or radiation therapy, if any), the surgeon may need to consider alternative strategies to deal with the emergency at hand. A nomogram including WHO functional status, albumin level and physiological evaluation has been suggested to guide in decision-making [44]. Notably, several alternative and minimal-invasive strategies can be adopted to handle the acute setting [45-47], sometimes bridging the emergency presentation to an elective and more controlled plan or, even avoiding major interventions at all in order to ensure relief of symptoms when no cure can be provided.

## Conclusions

Improving outcomes in emergency surgery for the geriatric population is a multifaceted task but has great clinical and health care system implications [48]. Organization of emergency care is important in order to improve outcomes. Evaluation of current practice is important to improve outcomes for the future. Acting on the identified deficits and finding new areas for research is important to improve outcomes in the elderly. However, we may not readily agree on what “outcomes” should be; should it be easy identifiers and easy picks, such as “mortality and morbidity”, or measures of function, satisfaction and even patients’ own perceptions?

Notably, among randomized controlled trials published in emergency medicine journals, only 5 trials (3%) specifically examined patients aged ≥60 years [49]. This emphasizes the need for better evidence and improved knowledge as the future emergency medicine health care resources will to a large degree be directed towards the elderly population.

We may agree on the vision to improve the emergency surgical care of elderly patients, yet lack the imperative to do so. Surgical trials have been few and far between and even more so in the case for emergency conditions requiring surgery [50]. Research for emergency conditions has particular legislative and organizational barriers to it, but also potential solutions that should be sought [51]. Collaboration across borders in order to arrive at better evidence of care is needed. National registries should be advocated for quality indicator performance, following the success of the Danish Clinical Register for Emergency Surgery [52]. Multicenter, international trials, such as the GlobalSurg project [53], are under way and can identify areas for future trials and will, hopefully, also give real-time data on delivery, process and outcome of emergency surgery in the elderly.

## Abbreviations

LMICs: Low- and middle-income countries
CT: Computed tomography
ASA: American Society of Anaesthesiologists score
UK: United Kingdom
US: United States
SPI: Simple Prognostic Index
WHO: World Health Organization
